# Epidemiological description and trajectories of patients with prostate cancer in Denmark: an observational study of 7448 patients

**DOI:** 10.1186/s13104-023-06599-2

**Published:** 2023-11-16

**Authors:** Victoria Blanes-Vidal, Ashkan Tashk, Manuella Lech Cantuaria, Rasmus Søgaard Hansen, Charlotte A. Poulsen, Mads H. Poulsen, Marie-Louise Krogh, Søren P. Sheikh, Esmaeil S. Nadimi

**Affiliations:** 1 Applied AI and Data Science Unit, The Maersk Mc-Kinney Møller Institute, University of Southern Denmark, Campusvej 55, 5230 Odense, Denmark; 2 Danish Centre for Clinical Artificial Intelligence (CAI-X), University of Southern Denmark, Odense University Hospital, Odense, Denmark; 3 Clinical Biochemistry Unit, Department of Clinical Research, Faculty of Health Sciences, University of Southern Denmark, Odense, Denmark; 4 Department of Urology, Odense University Hospital, Odense, Denmark; 5 Department of Urology, Hospital South West Jutland, Esbjerg, Denmark; 6 Department of Clinical Research, University of Southern Denmark, Odense, Denmark; 7 Blue-Cell Therapeutics, Ole Maaloes Vej 3, Copenhagen, Denmark

**Keywords:** Prostate cancer, Trajectory, Survival, Clinical data

## Abstract

**Objective:**

Identification of patients at high risk of aggressive prostate cancer is a major clinical challenge. With the view of developing artificial intelligence-based methods for identification of these patients, we are constructing a comprehensive clinical database including 7448 prostate cancer (PCa) Danish patients. In this paper we provide an epidemiological description and patients’ trajectories of this retrospective observational population, to contribute to the understanding of the characteristics and pathways of PCa patients in Denmark.

**Results:**

Individuals receiving a PCa diagnosis during 2008–2014 in Region Southern Denmark were identified, and all diagnoses, operations, investigations, and biochemistry analyses, from 4 years prior, to 5 years after PCa diagnosis were obtained. About 85.1% were not diagnosed with metastatic PCa during the study period (unaggressive PCa); 9.2% were simultaneously diagnosed with PCa and metastasis (aggressive-advanced PCa), while 5.7% were not diagnosed with metastatic PCa at first, but they were diagnosed with metastasis at some point during the 5 years follow-up (aggressive-not advanced PCa). Patients with unaggressive PCa had more clinical investigations directly related to PCa detection (prostate ultrasounds and biopsies) during the 4 years prior to PCa diagnosis, compared to patients with aggressive PCa, which may have contributed to the early detection of PCa.

**Supplementary Information:**

The online version contains supplementary material available at 10.1186/s13104-023-06599-2.

## Introduction

Prostate cancer (PCa) is the second most common diagnosed malignancy in men [1]. PCa is a heterogenous disease, with a wide range of disease pathogenesis, from asymptomatic and prolonged, to severe malignancy. Due to the rapid increase of digitalization in clinical settings, a huge amount of clinical data is being generated and collected everyday. Artificial intelligence (AI) can be used to extract knowledge from this complex data and assist clinical practice. In this context, the Danish Study of Prostate Cancer Markers (DANPRO) was initiated. The main purpose of DANPRO is to advance data-driven early identification of patients with aggressive PCa tumors by integrating historical trends and patterns of previous biochemical and clinical data, with PCa severity.

In this paper we provide an epidemiological description, information on diagnoses, and trajectories of this retrospective observational population including 7448 PCa patients (DANPRO dataset); to contribute to the understanding of the characteristics and pathways of PCa patients in Denmark.

## Methods

### Source data

We identified all patients living in the Region of Southern Denmark from 1st January 2004 to 31st December 2019, with a PCa diagnosis. (N = 18,529). We extracted data from different electronic health records (EHRs), on: (1) Basic patient data, including name, civil personal registration number, age, birthdate and death date; (2) Diseases, health-related conditions, operations and investigations, expressed based on the Health Care Classification System (SKS) [2], and (3) Information on all hospital laboratory analyses on blood samples, urine, cerebrospinal fluid and other bodily fluids, obtained from the Clinical Laboratory Information System Research Database (LABKA) and the BCC database [3, 4].

### Study population

Individuals with a PCa diagnosis between 1st January 2008 and 31st December 2014 (so called, indexing period) were identified from the source data, with the date of PCa diagnosis referred to as the index day. To ensure initial PCa diagnosis, individuals with PCa diagnosis during 2004–2007 were excluded from the analysis (N = 5005). We excluded all patients with index day during 2015–2019 (N = 6076), to warrant 5 years of follow-up after the first PCa diagnosis. The study population included 7448 patients. In clinical practice, when PCa is diagnosed, further clinical and imaging investigations are typically needed to rule-out the presence of metastasis. In 99% of our study population, these investigations took ≤ 36 days. After these 36 days, the PCa was labelled by the clinicians as being metastatic (M), not metastatic (NM) or presence of metastasis is unknown (UM) (Additional file 1).

For each patient, a pre-index and post-index period was defined (Additional file 2). The pre-index period is the 4-years period, before PCa diagnosis, from which we extracted descriptive data that characterize the patient before diagnosis, and that may act as potential predictors of PCa severity. The post-index period is the 5 years period after PCa diagnosis, from which we extracted descriptive data on the patient’s status, treatment, trajectory and progression after diagnosis.

### Data processing

In the dataset, the PCa diagnosis code of each patient often changes several times during the post-index period. This amalgam of trajectories was summarized by defining 7 subgroups of patients (Additional file 3). Each patient on the dataset was then labelled as belonging to one of the following classes of interest:

1. Patients with unaggressive PCa, i.e. patients who did not receive a diagnose of metastatic PCa at any time during the study period.
2. Patients with aggressive PCa (not advanced), i.e. patients who did not receive a diagnose of metastatic PCa on the index day (+ 36 days), but they were diagnosed with metastatic PCa at some point during the 5 years post-index period.
3. Patients with aggressive PCa (advanced), i.e. patients who received a diagnose of metastatic PCa on the index day + 36 days.

It must be noted that, in this categorization, the term “aggressive” does not refer to the cancer grade as assessed in a pathological analysis, but it is a mere descriptive term that refers to tumors that grow or spread quickly, and so metastasis is detected in ≤ 5 years.

### Statistical analyses

Statistical analyses were performed to compare demographic and clinical data among the groups: (1) Patients with unaggressive PCa, (2) Patients with aggressive PCa (not advanced) and (3) Patients with aggressive PCa (advanced). Differences in means were investigated by analysis of variance (ANOVA), followed by Tukey’s honestly significant difference test. Mood’s Median Test and post hoc test were used to investigate differences of medians. Rates were compared by tests of equal proportions. In all cases, a significance level of 0.05 was considered. All statistical analyses were performed in R statistical software.

## Results

### Disease frequency, patient trajectories and survival

**Fig. 1:**
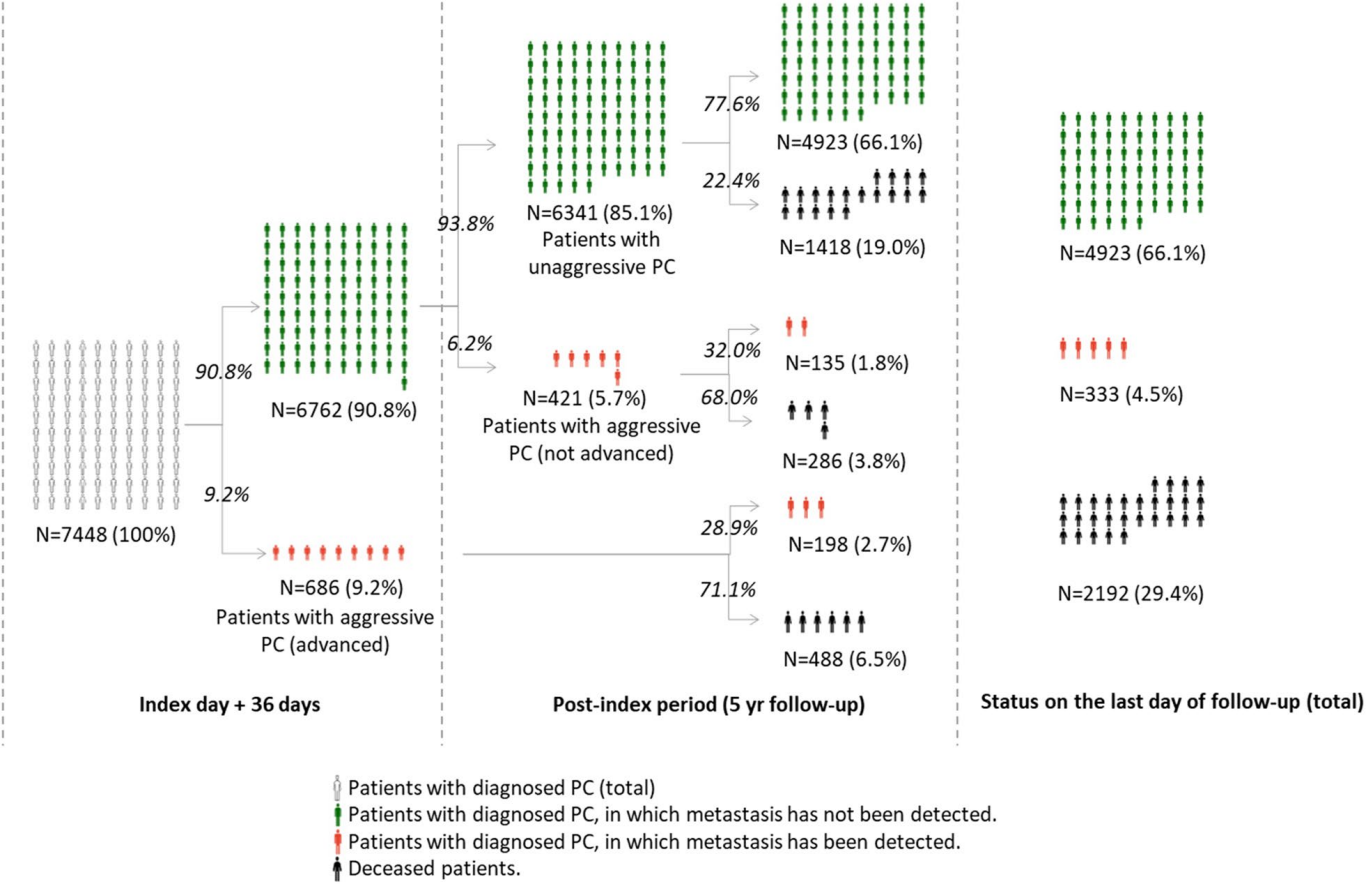
Disease frequency and patients’ trajectories in the study population. Percentages in parenthesis are percentages in respect to the original study population (N = 7448) and percentages in italics are percentages in respect to the previous step in the diagram

Out of the 7448 patients comprising the study population, 6341 patients (85.1%), did not receive a diagnose of metastatic PCa at any time during the study period; 686 patients (9.2%) were simultaneously diagnosed with PCa and metastasis during index day + 36 days, while 421 patients (5.7%) were not diagnosed with metastatic PCa at first (during index day + 36 days), but they were diagnosed with metastasis at some point during the subsequent 5 years follow up (Fig. 1).

Among the 6762 patients that were not diagnosed with metastatic PCa on index day (+ 36 days), the progression rate to metastasis during the 5 years follow-up period was 6.2%. The mean time to progression was 638 days (Additional file 4). The time to progression was < 1 year for 31% of the patients, 1–2 years for 25%, 2–3 years for 29%, 3–4 years for 15%, and 4–5 years for 0.2% of the patients.

A total number of 2192 patients died during the 5 years follow up, thus the 5-yr survival rate was 70.6%. The mortality rate in patients with unaggressive PCa (22.4%) was significantly lower than in patients with aggressive PCa, both not advanced (68.0%) and advanced (71.1%) (p < 0.05). The mortality rate was not significantly different among patients diagnosed with metastasis on the index day + 36 days (aggressive advanced PCa) compared to patients diagnosed with PCa afterwards (aggressive not advanced) (71.1% vs. 68%, p > 0.05).

The mean and median time between PCa diagnosis (index day) and death was 777 days and 712 days, respectively. This time was shortest (mean = 670 days, median = 572 days) among patients with aggressive advanced PCa, followed by patients with unaggressive PCa (mean = 787, median = 728 days) and patients with aggressive not-advanced PCa (mean = 895, median = 898 days) (p < 0.05).

### Age

**Fig. 2:**
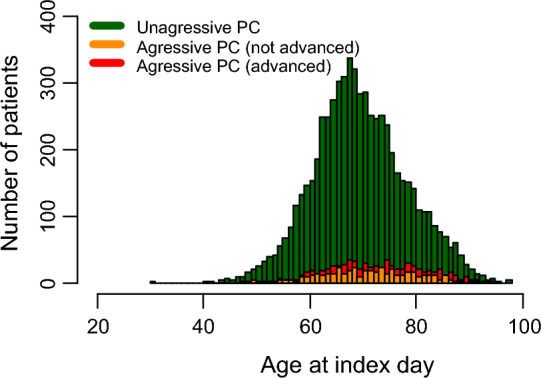
Age distribution for each of the categories, i.e. patients with unaggressive PC, patients with aggressive PC (not advanced) and patients with aggressive PC (advanced)

The age of PCa patients on the index day was on average 70.4 years (standard deviation = 8.8, median = 70.0 years, IQR = 11 years) (Fig. 2). The youngest PCa patient was 30 years old, and the eldest was 98 years old.

Patients with not aggressive PCa were on average younger than those with aggressive not-advanced PCa (70.1 years vs. 71.6 years, p < 0.05), and patients with aggressive advanced PCa (70.1 years vs. 73.0 years, p < 0.05). Besides, patients with aggressive not advanced PCa were on average younger than patients with aggressive advanced PCa (71.6 years vs. 73 years, p < 0.05). In terms of median age, patients with unaggressive PCa (median = 69 years) were younger than patients with aggressive PCa (median = 72–73 years) (p < 0.05).

### Clinical data

On average, each patient had 23.5 diagnoses and health-related conditions registered during the 4 years before PCa diagnosis, and about 94.3% had at least one code registered (Table 1). Out of the 175,376 codes, the number of unique codes (i.e. codes that are unrepeated, for each specific patient) was 49,264. Patients with aggressive advanced PCa had on average more unique diagnostic and health-related conditions registered during pre-index period, compared to patients with unaggressive PCa (7.18 vs. 6.52, p < 0.05).

**Table 1:** Clinical data on diagnoses, health-related conditions, operations, investigations and biochemistry analyses ^1^

		Unaggressive PC	Aggressive PC (not advanced)	Aggressive PC (advanced)
Diagnoses and health-related conditions^2^				
Total N_D_		147,743	10,759	16,874
Mean N_D_ per patient		23.3^a^	25.6^a^	24.6^a^
Mean N_D_ per patient (excluding outlier patients)^3^		14.8^a^	14.4^a^	16.2^b^
Median N_D_ per patient		12^a^	12^a^	14^b^
N_patients_ (% of patients) with ≥ 1 code		5972(94.2%)^a^	390(92.6%)^a^	664(96.8%)^b^
Total N_D_ (unrepeated for each patient)		41,373	2963	4928
Mean N_D_ per patient (unrepeated for each patient)		6.52^a^	7.04^ab^	7.18^b^
Median N_D_ per patient (unrepeated for each patient)		5^a^	5^a^	6^b^
Biochemistry analyses and biomarkers				
Total N_analyses_		43,263	3485	5731
Mean N_analyses_ per patient		6.8^a^	8.3^b^	8.4^b^
Mean N_analyses_ per patient (excluding outlier patients)^3^		4.0^a^	4.1^a^	5.1^b^
Median N_analyses_ per patient		3^a^	3^a^	3^a^
Total N_biomarkers_		545,347	45,057	71,569
Mean N_biomarkers_ per patient		86.0^a^	107^b^	104^b^
Mean N_biomarkers_ per patient (excluding outlier patients)^3^		44.8^a^	44.7^a^	63.2^b^
Median N_biomarkers_ per patient		25^a^	33^ab^	38^b^
Operations and investigations^4^				
Total N_OI_		485	34	55
Mean N_OI_ per patient		0.076^a^	0.081^a^	0.080^a^
Median N_OI_ per patient^5^		0^a^	0^a^	0^a^
N_patients_ (% of patients) with ≥ 1 code		144(2.27%)^a^	12(2.85%)^a^	19(2.77%)^a^
Total N_OI_ (unrepeated for each patient)		191	15	23
Mean N_OI_ per patient (unrepeated for each patient)		0.030^a^	0.036^a^	0.034^a^
Median N_OI_ per patient (unrepeated for each patient)^5^		0^a^	0^a^	0^a^
Specific prostate investigations				
Ultrasound	Mean	0.47^a^	0.38^b^	0.34^b^
	N (%) investigations	2954 (46.6%)^a^	161 (38.2%)^b^	234 (34.1%)^b^
	N (%) patients with ≥ 1	2415 (38.1%)^a^	146 (34.7%)^ab^	216 (31.5%)^b^
Biopsy	Mean	0.58^a^	0.44^b^	0.43^b^
	N (%) biopsies	3676 (58.1%)^a^	187 (44.2%)^b^	294 (42.9%)^b^
	N (%) patients with ≥ 1	3125 (49.3%)^a^	179 (42.5%)^b^	282 (41.1%)^b^

^1^ Different letters (a and b) indicate statistically significant differences between categories (unaggressive PC, aggressive PC not advanced and Aggressive PC advanced) at p-value < 0.05 using test of equal proportions (for rates), ANOVA and Tukey test (for means) and Mood’s Median Test and post hoc test (for medians)

^2^ N_D_ = Number of registered codes on diagnoses and health related conditions (starting with D in SKS)

^3^ Data lying outside Q3 + 1.5*IQR or Q1-1.5*IQR is considered as an outlier

^4^ N_OI_ = Number of registered codes on operations and investigations (starting with K and U in SKS)

^5^ Median equal to 0 is obtained, since > 50% of the values are 0

The database contains information on a total number of 52,479 analyses of bodily fluids, that were performed during the pre-index period, resulting in 661,973 biomarkers registered. The number of biochemistry analyses performed per patient, and individual biomarkers measured per patient during the pre-index period was significantly lower among patients with unaggressive PCa, compared to patients with aggressive advanced PCa (Table 1).

The number of operation and investigation codes per patient did not differ among the three patients’ categories (Table 1). However, the average number of ultrasound scannings of prostate, 4 years prior to PCa diagnosis was higher in the group of patients with unaggressive PCa, compared to the groups of patients with aggressive PCa forms (not advanced, 0.47 vs. 0.38, p < 0.05; and advanced, 0.47 vs. 0.34, p < 0.05). The percentage of patients that underwent at least one ultrasound investigation of prostate during the pre-index period, was significantly higher among patients with unaggressive PCa, compared to patients with aggressive advanced PCa forms (38.1% vs. 31.5%, p < 0.05). The average number of prostate biopsies was significantly higher in the group of patients with unaggressive PCa compared to the group of patients with aggressive PCa (not advanced, 0.58 vs. 0.44, p < 0.05; and advanced, 0.58 vs. 0.44, p < 0.05).

## Discussion

In this paper we have presented an overall summary of data currently included in the DANPRO database and performed statistical analyses for comparison among the three categories of interest.

### Challenges

We identified and overcame three main challenges:

1. *The large variety of clinical trajectories of PCa patients included in the dataset*: The dynamic and heterogeneous nature of the database hindered the definition of subcategories of patients. We overcame this challenge by defining 7 subgroups and 3 categories of interest, where all 7448 patients in the database could be allocated.
2. *The use of the diagnostic labelling “Unknown metastasis”*: This labelling, which is no longer in use in Denmark, introduces an additional difficulty when categorizing patients, specially in the case of patients who did not get a definite diagnosis in terms of “no metastasis” or “metastasis”, at any time during the 5-yr post-index period. The use of this labelling may be due to, e.g. patients with comorbidities or contraindications that prevent performing specific imaging tests, or patients who refuse further evaluation following a positive PCa diagnosis.
3. *The presence of females in the database*: Out of the 18,529 patients in the original (raw) database, we observed the presence of 341 female patients. A detailed examination of a sample of these clinical records, showed that this could be caused by errors in the use of diagnostic codes (e.g. women with breast cancer that were erroneously introduced in the database as PCa patients). The presence of transgender females in the EHR (i.e. assigned male at birth, but identified as females) should also be taken into account. The risk of prostate cancer in transgender women who are not on gender-affirming hormone therapy or surgery is the same as that in the cis male population [5].

### Strengths

Our study presents important strengths. First, we are among the first to collect and include in a PCa database, comprehensive data from an extensive period before initial PCa diagnosis, which, with the appropriate AI analysis, could be used for clinical decision making. Data during pre-index period revealed that patients with unaggressive PCa had a lower number of clinical data prior to PCa diagnosis, than patients with aggressive PCa. Although this can be partly explained by the fact that they were generally younger (possibly with less comorbidities) than patients with aggressive PCa, our study also revealed that patients with unaggressive PCa had a higher number of clinical investigations directly related to PCa detection (prostate ultrasounds and biopsies) before PCa diagnosis, compared to patients with aggressive PCa. Further analyses will allow to investigate whether the higher number of ultrasounds and biopsies in this group may have contributed to the early detection of PCa in these patients.

Another main strength of our study is the fact that it includes data from a large population (N = 7448). As studies on PCa with larger samples sizes are rare [6], we present highly relevant findings to depict the reality of PCa patients’ trajectories in the Danish healthcare system. Our study includes at present, data from the Region Southern Denmark, which, corresponds to 23% of the male population between 60 and 80 years old living in Denmark in 2011 [7]. The same methodology could be applied at national level to increase the sample size to about 20.800 patients.

### Limitations

Currently, our database does not include information on the histopathological differentiation of PCa (Gleason score or ISUP Gleason Grading Score) and/or Prostate Imaging-Reporting and Data System (PI-RADS). Besides, mortality rate is currently expressed in terms of “all-cause mortality”. Future work will focus on including histopathological and imaging information of the PCa at diagnosis; and obtaining information on the cause of death from the Danish register of Causes of Death [8], so that, mortality of each patient can be attributed to PCa disease, PCa treatment related comorbidities, or to other causes. Furthermore, the dataset, in its current form, does not include data on the doses and types of medications prescribed to the patients during the study period. Prescription data can contain useful clinical information on the PC patients, e.g. regarding comorbidities, which could further strengthen the database.

### Supplementary information

**Additional file 1:** SKS codes associated with Prostate Cancer and its metastasis.

**Additional file 2:** Indexing period, pre-index period and post-index period.

**Additional file 3:** Description of the 7 subgroups of patients.

**Additional file 4:** Mortality rate and time between index day and death.

## Data Availability

The datasets analyzed during the current study are not publicly available due to national act concerning personal privacy and rights, but are available from the corresponding author on reasonable request, and after approval from the Danish Data Protection Agency.
