# Supplementary material for: Epidemiological description and trajectories of patients with prostate cancer in Denmark: an observational study of 7448 patients
Source: BMC Res Notes. 2023 Nov 16;16:341. doi: 10.1186/s13104-023-06599-2 (PMC10655388; doi:10.1186/s13104-023-06599-2)
Supplement: Supplementary file 1 — Additional file 1: SKS codes associated with Prostate Cancer and its metastasis. [file 13104_2023_6599_MOESM1_ESM.docx]

**Additional file information**

**Additional file 1: SKS codes associated with Prostate Cancer and its metastasis**

| Table 1S. SKS codes associated with Prostate Cancer and its metastasis | | |
| --- | --- | --- |
| Code Names | Code Definitions in Danish | Code definitions in English |
| DC619x(Null,X,Y,Z)* | Prostatakræft | Prostate cancer |
| DC619M | Prostatakræft med metastaser | Prostate cancer with metastasis |
| AZCD40 | Ingen fjernmetastaser | No distant metastasis |
| AZCD41x (A, B, C)** | Fjernmetastaser | Distant metastasis |
| AZCD49 | Ingen klar oplysning om fjernmetastaser | No clear evidence for distant metastasis |

* X, Y, and Z stand for Local recurrence of prostate cancer, PSA recurrence after previously intended curative treatment without proven local recurrence or metastasis, and Castration-resistant prostate cancer, respectively.

** A, B, and C stand for no regional lymphnode, bone, and in the other organ metastases, respectively.
