# Supplementary material for: Epidemiological description and trajectories of patients with prostate cancer in Denmark: an observational study of 7448 patients
Source: BMC Res Notes. 2023 Nov 16;16:341. doi: 10.1186/s13104-023-06599-2 (PMC10655388; doi:10.1186/s13104-023-06599-2)
Supplement: Supplementary file 2 — Additional file 2: Indexing period, pre-index period and post-index period. [file 13104_2023_6599_MOESM2_ESM.docx]

**Additional file 2: Indexing period, pre-index period and post-index period.**


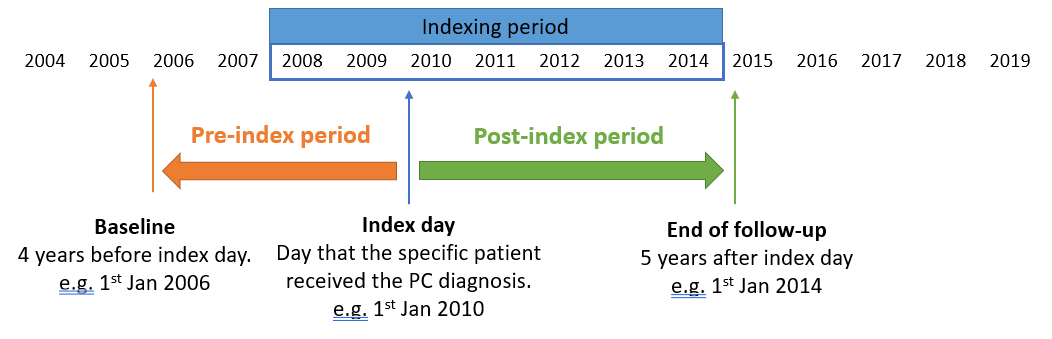


Figure S1. Indexing period (i.e. period of time in which we identified patients receiving a PC diagnosis) from 1st January 2008 and 31st December 2014 (i.e. 2922 index days) containing 7448 patients in total. For each patient, the index date is the date of PC diagnosis. Baseline was 4 years before index day (pre-index period) and the total follow-up period was 5 years after index day (post-index period).
