# Supplementary material for: Epidemiological description and trajectories of patients with prostate cancer in Denmark: an observational study of 7448 patients
Source: BMC Res Notes. 2023 Nov 16;16:341. doi: 10.1186/s13104-023-06599-2 (PMC10655388; doi:10.1186/s13104-023-06599-2)
Supplement: Supplementary file 3 — Additional file 3: Description of the 7 subgroups of patients. [file 13104_2023_6599_MOESM3_ESM.docx]

**Additional file 3: Description of the 7 subgroups of patients**

Subgroup 1. No-metastasis/no-metastasis (NM-NM): NM-NM are patients who were diagnosed with non-metastatic PCa on the index day + 36 days, and within the 5 years of follow up (post-index period), they never received a diagnose of metastatic PCa.

Subgroup 2. No-metastasis/metastasis (NM-M): Patients diagnosed with non-metastatic PCa on the index day + 36 days, but within the 5 years of follow up (post-index period), they receive a diagnose of metastatic PCa.

Subgroup 3. No-metastasis/Unknown-metastasis (NM-UM): patients who were diagnosed with non-metastatic PCa on the index day + 36 days; and within the 5 years of follow up (post-index period), they never receive a diagnose of metastatic or non-metastatic PCa.

Subgroup 4. Metastasis (M): Patients diagnosed with metastatic PCa on the index day + 36 days.

Subgroup 5. Unknown-metastasis/No-metastasis (UM-NM): Patients diagnosed with PCa that, on the index day + 36 days, it is unknown whether it is metastatic or non-metastatic; and within the 5 years of follow up (post-index period), they receive a diagnose of non-metastatic PCa.

Subgroup 6. Unknown-metastasis/Metastasis (UM-M): Patients diagnosed with PCa that, on the index day + 36 days, it is unknown whether it is metastatic or non-metastatic; and within the 5 years of follow up (post-index period), they receive a diagnose of metastatic PCa.

Subgroup 7. Unknown-metastasis/unknown-metastasis (UM-UM): Patients diagnosed with PCa that, on the index day + 36 days, it is unknown whether it is metastatic or non-metastatic; within the 5 years of follow up (post-index period), they never receive a diagnose metastatic or non-metastatic PCa.


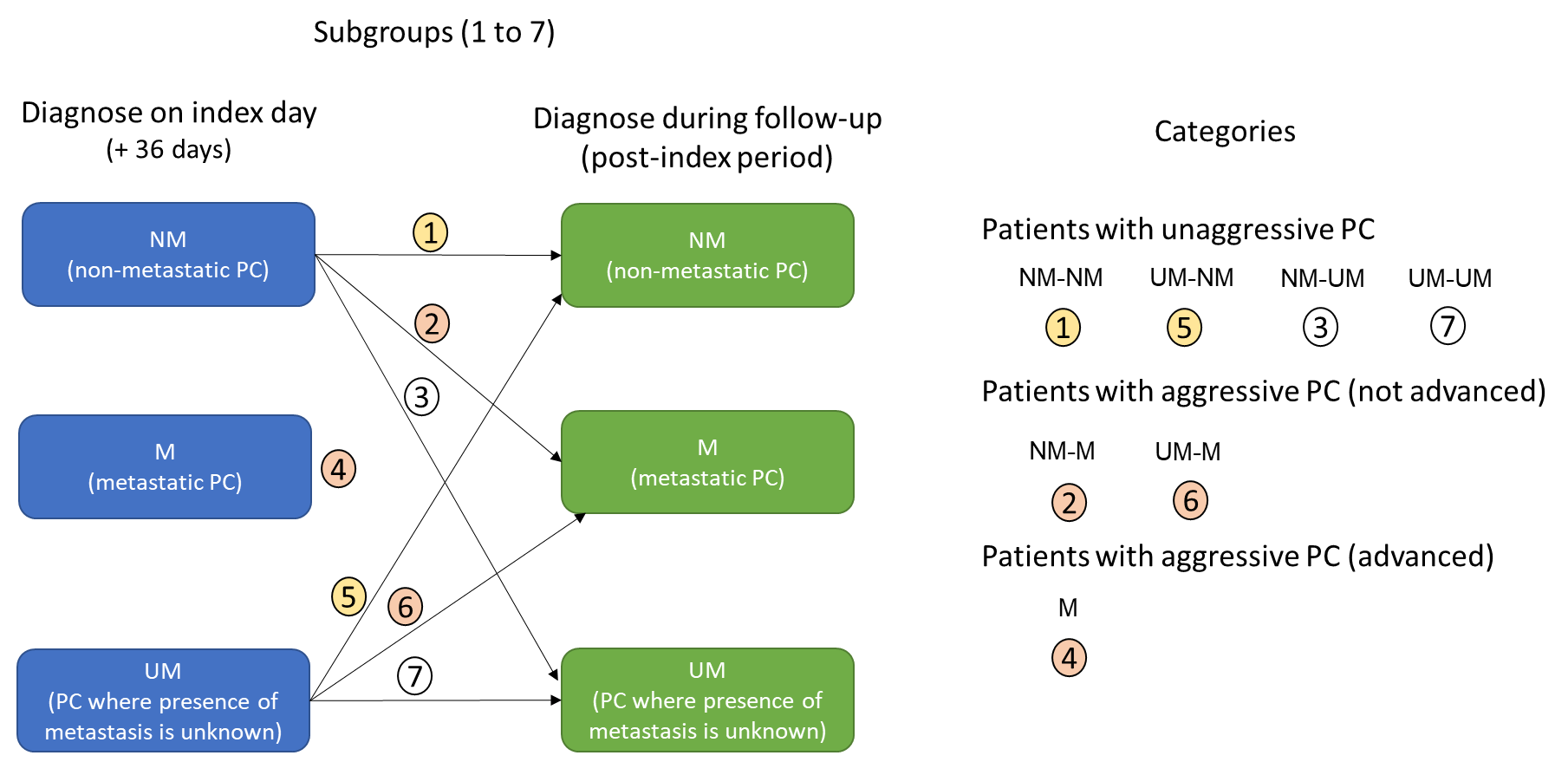


Figure S2. Patients’ subgroups (i.e. NM-NM (Subgroup 1), NM-M (Subgroup 2), NM-UM (Subgroup 3), M (Subgroup 4), UM-NM (Subgroup 5), UM-M (Subgroup 6), and UM-UM (Subgroup 7)); and categories of interest (i.e. patients with unaggressive PC, patients with aggressive PC (not-advanced) and patients with aggressive PC (advanced))

Patients in subgroups 3 (NM-UM) and 7 (UM-UM), did not get a definite diagnosis in terms of “no metastasis” or “metastasis”, at any time during the 5-yr post index period. In the case of patients with unknown PCa forms during follow-up period (Subgroup 3 and 7), we performed an exploratory analysis of 200 patients (100 patients per group), and after a detailed examination of the EHR, we concluded that patients belonging to Subgroup 3 (NM-UM) and Subgroup 7 (UM-UM) should also be considered as having an “unaggressive PCa”. When building the database, we followed the accepted approach used in clinical practice, in which the absence of a clinical test that confirms metastasis is considered as non-metastatic PCa. The acceptability of this approach was further confirmed when comparing the mortality rates among the subgroups that conform the category “unaggressive PCa” (groups 1, 3, 5 and 7), which generally not different from each other, and were strongly different from the mortality rates of the aggressive PCa subgroups (groups 2, 6 and 4).
