# Supplementary material for: Epidemiological description and trajectories of patients with prostate cancer in Denmark: an observational study of 7448 patients
Source: BMC Res Notes. 2023 Nov 16;16:341. doi: 10.1186/s13104-023-06599-2 (PMC10655388; doi:10.1186/s13104-023-06599-2)
Supplement: Supplementary file 4 — Additional file 4: Mortality rate and time between index day and death. [file 13104_2023_6599_MOESM4_ESM.docx]

**Additional file 4: Mortality rate and time between index day and death**

| Table 2S. Mortality rate and time between index day and death for the three patients categories^1^ | | | | | |
| --- | --- | --- | --- | --- | --- |
|  |  | Mortality during post-index period (5 years after index day) | | | |
| Patients categories | Total | N | Rate (%) | Time between index day and death, days | |
|  |  |  |  | Mean | Median |
| Unaggressive PC | 6341 | 1418 | 22.4^a^ | 787^a^ | 728^a^ |
| Aggressive PC (not advanced) | 421 | 286 | 68.0^b^ | 895^b^ | 898^b^ |
| Aggressive PC (advanced) | 686 | 488 | 71.1^b^ | 670^c^ | 572^c^ |
| ^1^Different letters indicate statistically significant differences between categories at p-value<0.05 using test of equal proportions (for rates), ANOVA and Tukey test (for means) and Mood’s Median Test and post hoc test (for medians). | | | | | |

| Table 3S. Mortality rate and time between index day and death for the seven subgroups^1^ | | | | | | |
| --- | --- | --- | --- | --- | --- | --- |
|  | Total | Mortality during post-index period (5 years after index day) | | | | |
|  |  | N | Rate (%) | Time between index day and death, days | | |
| Subgroup |  |  |  | Mean | STD | Median |
| NM-NM (Group 1) | 1998 | 482 | 24.1a | 644 | 542 | 542 |
| NM-M (Group 2) | 152 | 105 | 69.1c | 825 | 798 | 798 |
| NM-UM (Group 3) | 322 | 82 | 25.5ab | 964 | 983 | 983 |
| M (Group 4) | 686 | 488 | 71.1c | 670 | 572 | 572 |
| UM-NM (Group 5) | 1910 | 378 | 19.8b | 787 | 735 | 735 |
| UM-M (Group 6) | 269 | 181 | 67.3c | 936 | 940 | 940 |
| UM-UM (Group 7) | 2111 | 476 | 22.5ab | 902 | 869 | 869 |
| ^1^ Different letters between subgroups 1 to 7, indicate statistically significant differences at p-value<0.0023 (using the Bonferroni correction for multiple comparisons (for 21 comparisons, p-value < 0.05/21 = 0.0023) | | | | | | |
